# Coronary Stent Artifact Reduction with an Edge-Enhancing Reconstruction Kernel – A Prospective Cross-Sectional Study with 256-Slice CT

**DOI:** 10.1371/journal.pone.0154292

**Published:** 2016-04-29

**Authors:** Stéphanie Tan, Gilles Soulez, Patricia Diez Martinez, Sandra Larrivée, Louis-Mathieu Stevens, Yves Goussard, Samer Mansour, Carl Chartrand-Lefebvre

**Affiliations:** 1 Radiology, University of Montreal Medical Center (CHUM), Montreal, Canada; 2 University of Montreal Medical Center Research Center, Montreal, Canada; 3 Radiology, Centre Hospitalier Universitaire de Sherbrooke (CHUS), Sherbrooke, Canada; 4 Biostatistics, Pennington Biomedical Research Center, Bâton Rouge, Louisiana, United States of America; 5 Cardiac Surgery, University of Montreal Medical Center (CHUM), Montreal, Canada; 6 Electrical Engineering, Ecole Polytechnique de Montréal, Montreal, Canada; 7 Cardiology, University of Montreal Medical Center (CHUM), Montreal, Canada; University of Bologna, ITALY

## Abstract

**Purpose:**

Metallic artifacts can result in an artificial thickening of the coronary stent wall which can significantly impair computed tomography (CT) imaging in patients with coronary stents. The objective of this study is to assess in vivo visualization of coronary stent wall and lumen with an edge-enhancing CT reconstruction kernel, as compared to a standard kernel.

**Methods:**

This is a prospective cross-sectional study involving the assessment of 71 coronary stents (24 patients), with blinded observers. After 256-slice CT angiography, image reconstruction was done with medium-smooth and edge-enhancing kernels. Stent wall thickness was measured with both orthogonal and circumference methods, averaging thickness from diameter and circumference measurements, respectively. Image quality was assessed quantitatively using objective parameters (noise, signal to noise (SNR) and contrast to noise (CNR) ratios), as well as visually using a 5-point Likert scale.

**Results:**

Stent wall thickness was decreased with the edge-enhancing kernel in comparison to the standard kernel, either with the orthogonal (0.97 ± 0.02 versus 1.09 ± 0.03 mm, respectively; p<0.001) or the circumference method (1.13 ± 0.02 versus 1.21 ± 0.02 mm, respectively; p = 0.001). The edge-enhancing kernel generated less overestimation from nominal thickness compared to the standard kernel, both with the orthogonal (0.89 ± 0.19 versus 1.00 ± 0.26 mm, respectively; p<0.001) and the circumference (1.06 ± 0.26 versus 1.13 ± 0.31 mm, respectively; p = 0.005) methods. The edge-enhancing kernel was associated with lower SNR and CNR, as well as higher background noise (all p < 0.001), in comparison to the medium-smooth kernel. Stent visual scores were higher with the edge-enhancing kernel (p<0.001).

**Conclusion:**

In vivo 256-slice CT assessment of coronary stents shows that the edge-enhancing CT reconstruction kernel generates thinner stent walls, less overestimation from nominal thickness, and better image quality scores than the standard kernel.

## Introduction

With technological advances in computed tomography (CT) systems, noninvasive diagnostic capability of coronary CT angiography (CTA) for in-stent restenosis has gradually improved [1–5]. However, metallic artifacts remain a significant and specific drawback in CTA imaging of coronary stents [3]. These artifacts create an artificial thickening of the stent struts with apparent in-stent luminal narrowing, with severe impairment to lumen assessment [4, 6, 7].

Specific CT image reconstruction kernels have been introduced in order to reduce these stent metallic artifacts. Sharp kernels offered by CT vendors for coronary stent imaging preserve higher spatial frequencies, however at the expense of greater noise [8].

Most studies performed to assess the effect of sharp kernels in coronary CT stent imaging were done *in vitro* [9–17]. Only few *in vivo* studies have quantitatively evaluated the effect of CT kernels on coronary stent artifacts [18–20]. The objective of this clinical prospective 256-slice CT study is to provide an *in vivo* assessment of the effect of an edge-enhancing CT reconstruction kernel on the visualization of stent wall and lumen and on image quality scores as compared to a standard kernel.

## Material and Methods

### Study Design

This is a cross-sectional study with repeated measure design and blinded observers, which prospectively recruits patients with coronary stents, and compares the effect of two CT reconstruction kernels on stent wall thickness, as well as on visual and quantitative parameters of stent image quality.

### Study Patients

The local Institutional Review Board of the University of Montreal Medical Center approved the protocol, and all subjects provided written informed consent.

A total of 24 consecutive patients (mean age 61.2 +/- 9.6 (standard deviation, SD) yo; 18 males, 59.9 +/- 9.7 yo; 6 females, 65.2 +/- 8.8 yo) were prospectively enrolled. All patients had at least one stent implanted in the left main coronary artery (LMA) and / or the proximal right coronary artery (RCA) to treat de novo coronary stenoses during the previous month. Besides these inclusion criteria, patients with additional distal coronary stents and / or former additional stents could also be enrolled.

Patients were ineligible for the study if they were < 18 or > 85 yo, had hypersensitivity to iodine-containing compounds, decreased renal function (glomerular filtration rate < 50 mL/min/1.73 m^2^)), or inability to give informed consent.

### CT Imaging

#### Patient preparation

Patients were given 50–100 mg of metoprolol orally 45–60 minutes prior to CT if heart rate was > 60 beats per minute (bpm), and 0.4 mg of nitroglycerin sublingually, in absence of contraindications.

#### CT image acquisition

A 256-slice CT scanner was used (Brilliance iCT, Philips Healthcare, Cleveland, OH, USA). Collimation was 0.625-mm, matrix 512 x 512, field-of-view 250 mm, scan voltage 120 kV, and gantry rotation 270ms. Prospective ECG-gating was used for all patients, targeting a diastolic phase at 75% of R-R cycle, with a phase tolerance of ± 5% (intracycle buffer). Filtered back projection was used for image reconstruction.

#### Injection parameters

The contrast agent was injected at a flow rate of 5ml/sec, using iodixanol (320 mg I/mL, Visipaque 320, GE Healthcare Canada Inc., Mississauga, Ontario, Canada). The following protocol was used: 80 ml of 100% contrast agent, followed by 55 ml of 40% contrast agent and 60% saline, finally 40 ml of 100% saline.

#### CT image reconstruction and postprocessing

Axial reconstruction was done with a slice thickness of 0.8 mm (mean increment 0.4 mm), using two kernels: i) a medium-smooth kernel (XCB, Philips Healthcare, Cleveland, OH, USA), and ii) an edge-enhancing kernel (XCD, Philips Healthcare, Cleveland, OH, USA). For the XCB kernel, the 50% modulation transfer function (MTF) is 3.7 line pairs (lp) / cm. For the XCD kernel, the 50% MTF is 4.0 lp / cm. Images were then transferred to a thin client postprocessing system (Aquarius Intuition version 4.4.11, TeraRecon Headquarters, Forster City, CA, USA).

The coronary segments were defined as reported in the American College of Cardiology/American Heart Association guidelines for coronary angiography [21].

#### Radiation dose

The effective radiation dose of CT angiography was estimated by the product of the dose-length product (DLP), as indicated on the dose report of the CT scanner, and a conversion coefficient for the chest (k = 0.014 mSv*mGy−1*cm−1).

### CT image analysis

Two independent observers blinded to each other and to the reconstruction kernel performed quantitative image analysis. Observers no 1 and no 2 (CCL, GS) were board-certified radiologists (15 and 22-year experience in cardiovascular CT, respectively). The order of kernel presentation for each stent for both observers was generated by randomization (Heads or Tails 3D, version 1.2, iPhone platform). To maintain observer blinding as to the kernels, manipulation of the image data sets was performed by an independent research assistant, unaware of the characteristics of the kernels and the study objectives, and not involved in subsequent study evaluations. A fixed window setting was used for assessment of all stents (window width: 1500 Hounsfield units (HU); window centre: 300 HU), at maximal image magnification.

To quantify the artificial thickening of the stent walls created by the stent blooming artifacts, two methods were used, involving the measurement of the averaged stent orthogonal wall thickness or averaged circumference wall thickness.

#### Stent orthogonal diameter and circumference measurements

**Fig 1:**
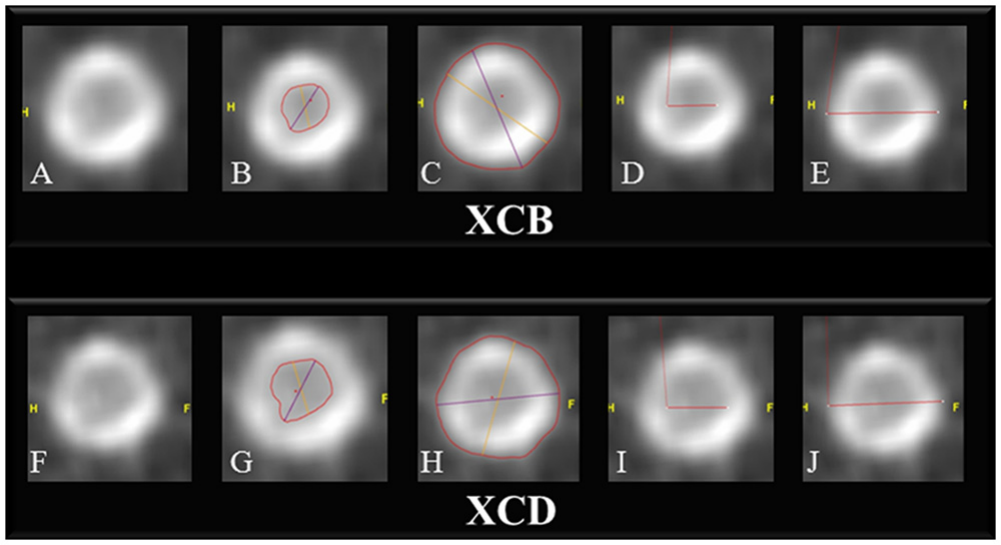
Method of quantitative stent wall thickness measurement. Coronary stent reformation in short axis perpendicular to centerline. Upper row (A–E), images after reconstruction with a medium-smooth kernel (XCB, Philips Healthcare, Cleveland, OH, USA); lower row, (F–J), images after reconstruction with an edge-enhancing kernel (XCD, Philips Healthcare, Cleveland, OH, USA). Internal (B, G) and external (C, H) stent areas measured using a semi-automatic segmentation software, with manual correction when needed (circumference thickness method). Internal (D, I) and external (E, J) stent diameters measured using manual placement of an electronic caliper for distance measurements (orthogonal thickness method).

Methodology had to ensure that measurements for a given stent were performed in the same location for both kernels and both observers. A short-axis reformation orthogonal to the stent centerline was created in the stent at a 3-mm long-axis distance from the origin of the stent. All measurements were then obtained at this location. In case of important calcifications, this location could be slightly modified from the predetermined 3-mm distance, and then used for both kernels. The first observer noted the position of the short-axis reformation plane and the second observer was asked to use this same position for measurements. The average orthogonal stent wall thickness was evaluated by measuring the internal and external stent diameters using manual placement of an electronic caliper for distance measurements. The average circumference stent wall thickness was determined by delineation of the internal and external stent areas using a semi-automatic segmentation system for area measurements, with manual correction when needed (Fig 1).

#### Stent wall thickness calculation

Stent wall thickness was calculated with the orthogonal thickness method and the circumference method, using the following eqs 1 and 2, respectively:

$$\text{Orthogonal stent wall thickness}=\frac{\text{stent external diameter}-\text{stent internal diameter}}{2}$$ (1)

$$\text{Circumference stent wall thickness}=\sqrt{\frac{{\text{CSA}}_{\text{ext}}}{\pi }}-\sqrt{\frac{{\text{CSA}}_{\text{int}}}{\pi }}$$ (2)

Where

CSA_ext_: cross-sectional area from external stent circumference;

CSAint: cross-sectional area from internal stent circumference.

#### Visual assessment of image quality

Assessment of image quality was performed in short-axis view, using a 5-point Likert scale according to the evaluation of the stent lumen and artifacts, quality of stent contour delineation, and general image quality (1, nondiagnostic: lumen non evaluable; 2, poor: poor lumen evaluation, severe stent artifacts; 3, moderate: lumen partially visible, moderate stent artifacts; 4, good: good lumen visibility, slight artifacts; 5, excellent: lumen distinctly visible, no visible artifacts).

#### Quantitative assessment of image quality

A third observer (ST) assessed the image noise, signal to noise ratio (SNR) and contrast to noise ratio (CNR) of both kernels in all stents. Mean in-stent lumen attenuation was obtained from five regions of interest (ROI) placed on a short axis reformation of each stent. Mean background noise was obtained using the mean standard deviation of five ROIs placed in the air surrounding the patient. In-stent lumen SNR was determined by dividing the mean attenuation value of the in-stent ROIs by the image noise. For CNR, the mean attenuation from five ROIs placed on the left ventricular lateral myocardial wall was used. In-stent CNR was determined by dividing the difference between the mean attenuation obtained from the myocardial ROIs and the mean attenuation obtained from the in-stent ROIs, by the image noise. The attenuation of the stent walls was not measured.

### Statistical analysis

Continuous data were expressed as mean value ± SD, and categorical data as proportions.

The association between kernel and stent wall thickness was analyzed with linear mixed models, with random effects on observer and on patients to account for patient clustered observations. The analysis of the association between kernel and image quality score was done using a proportional odds mixed model, with blinding of the biostatistician (SL) to the type of kernel.

Interobserver concordance for stent wall thickness (continuous variable) was assessed with the intraclass correlation coefficient (ICC) for absolute concordance of unique measurements, using a two-factor mixed model. An ICC <0.40 implied poor agreement; 0.40–0.59, fair agreement; 0.60–0.74, good agreement; 0.75–1.00, excellent agreement [22]. Interobserver agreement for the scores of image quality (ordinal variable) was assessed with the Cohen's kappa statistic with linear weighting for ordinal variables [23], and with the Cohen's kappa statistic for nominal variables, after dichotomization [24]. A kappa value < 0.20 implied poor agreement; 0.21–0.40, fair agreement; 0.41–0.60, good agreement; 0.61–0.80, very good agreement; and 0.81–1.00, excellent agreement [25].

For within-patient comparison of continuous variables showing normal distribution of paired differences, the paired t-test was used. Otherwise, the Wilcoxon signed-rank test was used. Pearson χ^2^ test was used to compare categorical variables. A two-tailed p value <0.05 was considered statistically significant. Statistical analyses were performed using SPSS (SPSS version 20, Inc, Chicago, Illinois) and R (R version 3.1.1) [26]. Weighted kappa coefficients were calculated with specific SPSS command lists [27, 28], as well as with vassarstat.net online calculator [29].

## Results

### Patient population

The 24 patients had a total of 71 stented coronary artery segments: 38 (53.5%) on the left coronary artery, and 33 (46.5%) on the RCA. All stents were patent on the basis of CTA. Patient demographics, scanning parameters and stent distribution are described in Tables 1 and 2. For the 71 stented segments, information on manufacturer and stent type was available for 55 stents (Table 3).

**Table 1:** Patient characteristics and scan-related parameters.

**Patient characteristics (N = 24 patients)**	
Men / women	18 / 6
Age (years) (mean ± SD)	61.2 ± 9.6
BMI (kg/m2) (mean ± SD)	29.6 ± 6.7
Smoker (n*, %)	11 (46)
Hypertension (n, %)	13 (54)
Hypercholesterolemia (n, %)	17 (71)
Diabetes (n, %)	4 (17)
Stented segments / patient (mean ± SD)	2.96 ± 1.27
Time interval between stent implantation and CT (days) (mean ± SD) (range)	17.3 ± 3.4 (11–22)
**Scan-related parameters**	
Mean heart rate during scan (bpm) (mean ± SD)	54.6 ± 7.8
Heart rate variability ** during scan (bpm) (mean ± SD)	2.7 ± 6.9
Prescan betablocker administration, n (%)	14 (58.3)
Prescan nitroglycerin administration, n (%)	19 (79.2)
Contrast agent (ml) (mean ± SD)	96.0 ± 10.5
DLP *** (mGy-cm) (mean ± SD)	380.2 ± 65.0
Effective dose **** (mSv) (mean ± SD) (range)	5.3 ± 0.9 (3.7–6.9)

Standard deviation (SD); body mass index (BMI); beats per minutes (bpm)

*: n, number of patients

**: The standard deviation of the heart rate in a given patient was the parameter used for heart rate variability measurement.

***: DLP, dose-length product for MDCT angiography

****: The effective radiation dose was estimated by the product of the DLP and a conversion coefficient for the chest (k = 0.014 mSv*mGy−1*cm−1). Prospective ECG-gating was used in all patients.

**Table 2:** Coronary artery stent segmental distribution* (N = 24 patients).

Total number of segments with stents	71
**Left coronary artery (n, %)**	
LMA	10 (14.1)
Proximal LAD	7 (9.9)
Mid LAD	4 (5.6)
Distal LAD	3 (4.2)
Proximal CX	7 (9.9)
Mid CX	5 (7.0)
1^st^ obtuse marginal	2 (2.8)
**Right coronary artery (n, %)**	
Proximal RCA	12 (16.9)
Mid RCA	11 (15.4)
Distal RCA	9 (12.7)
PDA	1 (1.4)

Left main coronary artery (LMA); Left anterior descending artery (LAD); Left circumflex artery (CX); Right coronary artery (RCA); Posterior descending artery (PDA)

**Table 3:** Types of stents*.

Name	Manufacturer **	Materiel	Coating	Nominal internal diameter (mm)								Nominal strut thickness (mm)
				2.0	2.5	2.8	3.0	3.5	4.0	4.5	5.0	
Cypher	Cordis	Stainless steel	Sirolimus ***	0	0	0	0	1	0	0	0	0.14
Xience Prime	Abbott	CoCr ****	Everolimus ***	0	0	0	6	3	0	0	0	0.08
Xience V	Abbott	CoCr	Everolimus ***	1	4	1	7	12	1	0	1	0.08
Vision	Abbott	CoCr	None	1	1	0	1	2	2	0	0	0.08
Mini Vision	Abbott	CoCr	None	0	0	1	0	0	0	0	0	0.08
Multi Link Ultra	Abbott	Stainless steel	None	0	0	0	0	0	0	1	1	0.08
Endeavor	Medtronic	CoCr	Zotarolimus ***	0	0	0	2	0	0	0	0	0.09
Integrity	Medtronic	CoCr	None	0	1	1	0	0	2	0	0	0.09
Prokinetic	Biotronik	CoCr	None	0	0	0	1	0	0	0	0	0.06
Orsiro	Biotronik	CoCr	Limus	0	1	0	0	0	0	0	0	0.06

* Among 71 stented coronary artery segments, the stent type is known in 55 segments.

**: Cordis Corporation, Miami Lakes, FL; Abbott Laboratories, Abbott Park, Illinois; Medtronic Minneapolis, Minnesota; Biotronik, Bülach, Switzerland

***: Drug-eluting stent

****: Cobalt—chromium alloy

### Quality control of postprocessing

All 71 (100%) stented segments were successfully evaluated by the two observers. Mean distances from the origin of the stents used for the short-axis reformation were similar for the medium-smooth (XCB) and edge-enhancing (XCD) kernels, ie 3.32 ± 1.00 and 3.33 ± 1.00 mm for observer 1 (p = 0.54) and 3.04 ± 0.06 and 3.05 ± 0.12 mm for observer 2 (p = 0.78), respectively.

### Stent wall thickness data

Nominal wall thickness of the stents is described in Table 3. Table 4 shows that the mean stent wall thickness as measured with CT varies from 0.89 ± 0.17 to 1.43 ± 0.17 mm.

**Table 4:** Stent CT measurements (71 stented segments).

	Observer 1	Observer 2
Stent wall thickness—orthog. th. method*, kernel XCB (mean, SD) (mm)	1.22 ± 0.24	0.97 ± 0.33
Stent wall thickness—circumf. method**, kernel XCB (mean, SD) (mm)	1.43 ± 0.17	1.00 ± 0.22
Stent wall thickness—orthog. th. method, kernel XCD (mean, SD) (mm)	1.05 ± 0.17	0.89 ± 0.17
Stent wall thickness—circumf. method, kernel XCD (mean, SD) (mm)	1.31 ± 0.19	0.95 ± 0.19
Thickness overestimation—orthog. th. method, kernel XCB (mean, SD) (mm)	1.14 ± 0.25	0.85 ± 0.19
Thickness overestimation—circumf. method, kernel XCB (mean, SD) (mm)	1.36 ± 0.18	0.90 ± 0.23
Thickness overestimation—orthog. th. method, kernel XCD (mean, SD) (mm)	0.96 ± 0.16	0.81 ± 0.17
Thickness overestimation—circumf. method, kernel XCD (mean, SD) (mm)	1.23 ± 0.20	0.88 ± 0.18

Stent wall thickness overestimation: manufacturer nominal wall thickness—wall thickness as measured with CT (55 stented segments)

Standard deviation (SD)

* orthog. th. method: orthogonal thickness method

**: circumf. method: circumference method

#### Interobserver agreement—ICCs

Interobserver agreement showed ICC values of -0.052 (95% confidence interval (CI), -0.215–0.134) and 0.219 (95% CI, -0.034–0.445) for stent wall thickness using the orthogonal thickness method with the XCB and XCD kernels, respectively; and values of 0.067 (95% CI, -0.059–0.227) and 0.116 (95% CI, -0.075–0.339) using the circumference method with the two kernels, respectively.

#### Stent wall thickness according to kernel attribution

Stent wall thickness as measured with the XCD kernel was significantly inferior than with the XCB kernel, either with the orthogonal method (0.97 ± 0.02 versus 1.09 ± 0.03 mm, respectively; p < 0.001) or with the circumference method (1.13 ± 0.02 versus 1.21 ± 0.02 mm, respectively; p = 0.001).

#### Stent wall thickness CT overestimation

For the 55 stents with known manufacturer parameters, the mean overestimation of CT measurement in comparison to the nominal stent wall thickness varied from 0.81 ± 0.17 to 1.36 ± 0.18 mm (Table 4).

**Fig 2:**
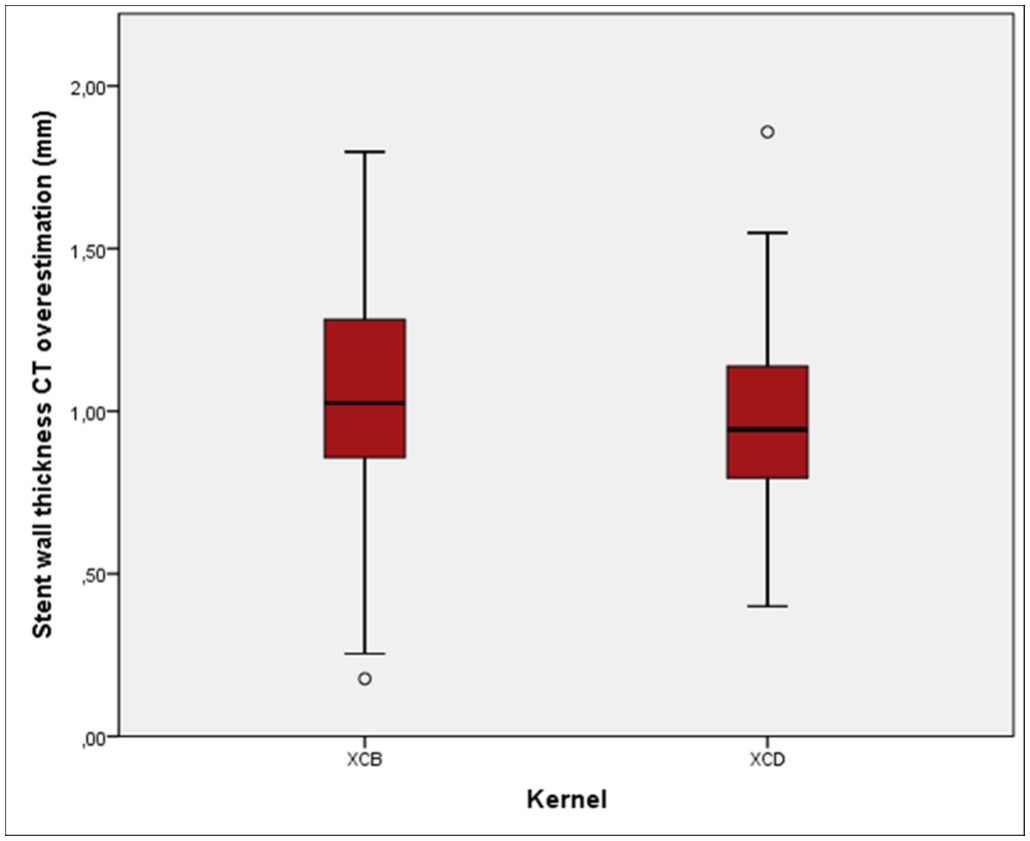
Stent wall thickness overestimation with smooth and sharp kernels. Box plots: Stent wall thickness overestimation using the XCD kernel was significantly less than with the XCB kernel (p < 0.005). In this figure, merged data is used for both diameter and circumference methods, as well as for both observers no 1 and 2.

There was significantly less overestimation with the XCD kernel than the XCB kernel, both with the orthogonal (0.89 ± 0.19 versus 1.00 ± 0.26 mm, respectively; p < 0.001) and the circumference (1.06 ± 0.26 versus 1.13 ± 0.31 mm, respectively; p = 0.005) methods (Fig 2).

### Visual assessment of image quality

Interobserver agreement showed weighted kappa values of 0.07 (95% CI, 0.00–0.20) and 0.03 (95% CI, 0.00–0.15) for visual scores using the XCB the XCD kernels, respectively. After dichotomization of scores (scores 1–3 versus 4–5), kappa values were 0.13 (95% CI, -0.06–0.33) and 0.18 (95% CI, -0.04–0.41), for the XCB and XCD kernels, respectively.

**Fig 3:**
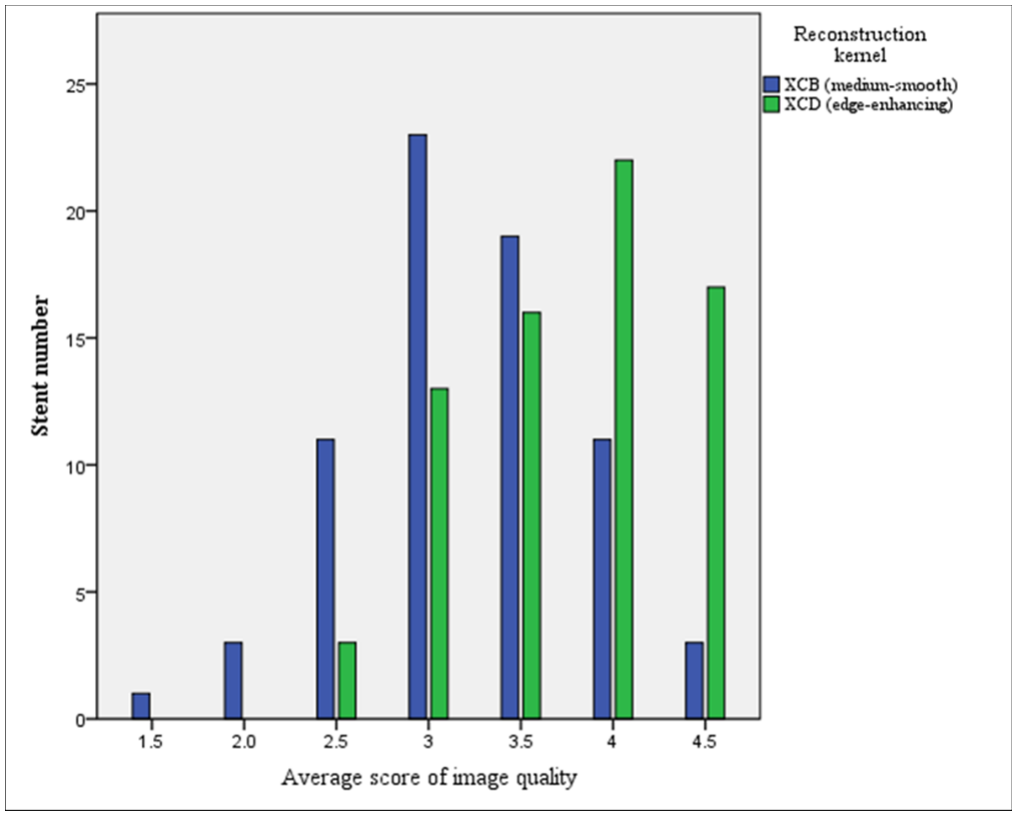
Qualitative stent image quality with smooth and sharp kernels. Average scores of visual assessment of image quality in 71 stented coronary artery segments. Distribution of average scores of visual assessment of image quality in 71 stented segments, as evaluated with 256-slice MDCT and prospective ECG-gating, after image reconstruction with medium-smooth (XCB) and edge-enhancing (XCD) reconstruction kernels. In this figure, each score is the average of the visual score of the two independent observers 1 and 2 for a given stent.

**Fig 4:**
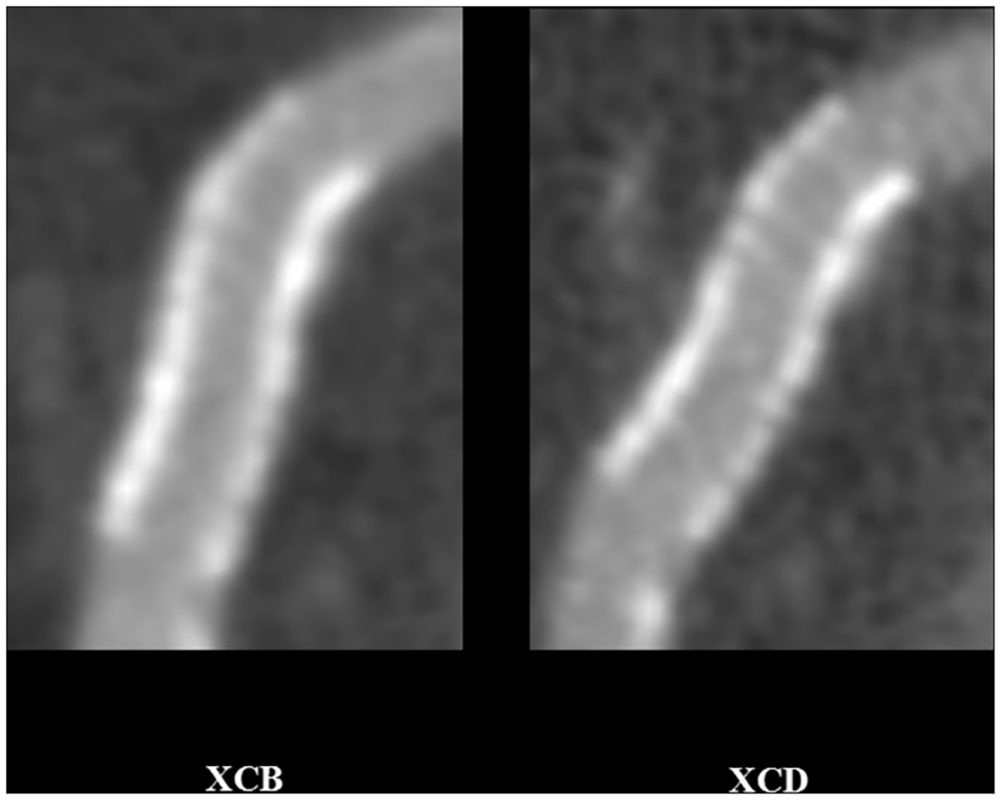
Reduced stent blooming artifacts and improved strut definition with sharp (XCD) in comparison to smooth (XCB) kernel. 77-year-old female, right proximal coronary artery patent bare-metal stent (Abbott Vision, length 15 mm, diameter 4 mm, nominal thickness 0.08 mm). 256-slice CT acquisition with prospective ECG-gating, and image reconstuction with a medium-soft (XCB, left) and edge-enhancing (XCD, right) reconstruction kernels, multiplanar reformat. Window width, 1500 HU; window centre: 300 HU, for both kernels. For observer 1, stent wall thickness for the XCB and XCD kernels with the orthogonal thickness method was 1.44 mm and 1.24 mm, and 1.66 mm and 1.58 mm with the circumference method, respectively. Image quality scores for observer 1 were 3 and 4, respectively. For observer 2, stent wall thickness for the XCB and XCD kernels with the orthogonal thickness method was 0.89 mm and 1.15 mm, and 1.20 mm and 0.93 mm with the circumference method, respectively. Image quality scores for observer 2 were 3 and 4, respectively.

**Fig 5:**
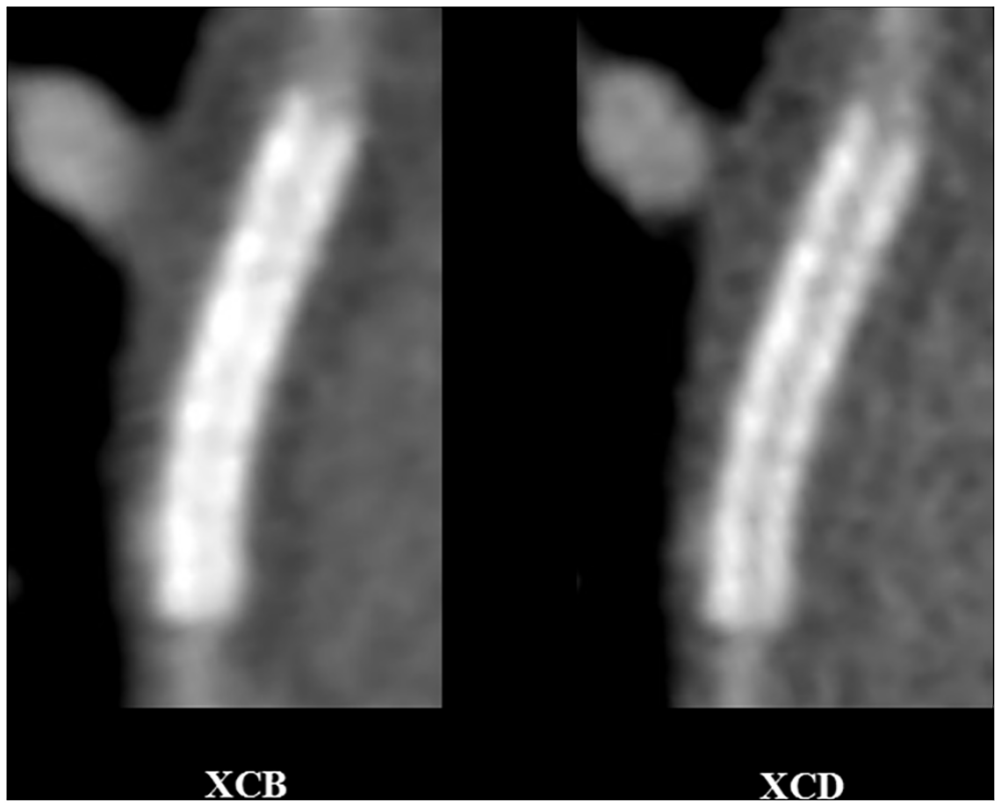
Reduced stent blooming artifacts and improved strut definition with sharp (XCD) in comparison to smooth (XCB) kernel. 69-year-old female, first obtuse marginal artery stent. 256-slice CT acquisition with prospective ECG-gating, and image reconstuction with a medium-soft (XCB, left) and edge-enhancing (XCD, right) reconstruction kernels, multiplanar reformat. Window width, 1500 HU; window centre: 300 HU, for both kernels. For observer 1, stent wall thickness for the XCB and XCD kernels with the orthogonal thickness method was 1.29 mm and 1.05 mm, and 1.26 mm and 1.26 mm with the circumference method, respectively. Image quality scores for observer 1 were 3 and 4, respectively. For observer 2, stent wall thickness for the XCB and XCD kernels with the orthogonal thickness method was 0.97 mm and 0.71 mm, and 0.82 mm and 0.83 mm with the circumference method, respectively. Image quality scores for observer 2 were 2 and 3, respectively.

More stented segments were associated with scores 4 (good) and 5 (excellent) using the XCD kernel: 53 (53/71, 74.6%) and 43 (43/71, 60.6%) for observers 1 and 2 respectively, in comparison with the XCB kernel: 17 (17/71, 23.9%) and 36 (36/71, 50.7%) for observers 1 and 2, respectively (p < 0.001 and p = 0.075 for observers 1 and 2, respectively) (Figs 3–5).

Higher mean scores of stent image quality were observed with the XCD (3.94 ± 0.75 and 3.58 ± 0.89 for observers 1 and 2, respectively) than with the XCB kernel (2.99 ± 0.75 and 3.44 ± 0.97, respectively; odds ratio (OR) 3.71, 95% CI 2.33–5.92; p < 0.001) (Fig 3). Proximal stents (LMA and proximal RCA) were also associated with higher scores (OR 2.21, 95% CI 1.38–3.56; p = 0.001). Heart rate did not influence image quality scores (OR 1.00, 95% CI 0.96–1.04; p = 0.875).

### Quantitative assessment of image quality (attenuation, noise, SNR, CNR)

The XCD kernel was associated with lower in-stent lumen attenuation (428.4 ± 96.0 HU, 471.3 ±121.2 HU, respectively), SNR (12.8 ± 5.9, 19.5 ± 12.7, respectively) and CNR (9.9 ± 4.8, 15.4 ± 10.6, respectively), as well as higher background noise (37.6 ± 12.2 HU, 31.1 ± 12.9 HU, respectively) (all, p < 0.001), in comparison to the XCB kernel.

## Discussion

This clinical 256-slice CT study shows that imaging of coronary stents with an edge-enhancing reconstruction kernel results in lower stent wall thickness, less stent wall overestimation from nominal thickness, and better visual scores of in-stent lumen image quality in comparison to a medium-smooth kernel.

A recent meta-analysis of coronary stent imaging with 64-slice scanners has reported a pooled sensitivity and specificity of 86% and 93% respectively, for in-stent restenosis [3]. However, the mean rate of nonassessable stents remained as high as 9% (range 0 to 42%) [3]. Blooming artifacts are among of the most detrimental and specific CT stent imaging problem, causing severe impairment to in-stent restenosis detection [4]. The blooming effect results from an X-ray beam hardening phenomenon. Absorption of low-energy photons causes a global increase of the energy spectrum of the X-ray beam as it crosses highly attenuating structures such as metallic stents. The consequence is an artificial thickening of the stent struts, with apparent in-stent luminal narrowing [6, 7]. Partial volume effect also contributes to the blooming effect [7].

Most studies of the effect of edge-enhancing kernels in coronary stent CT imaging have been done *in vitro* [9–17, 30, 31], either with static [9, 10, 15, 16, 30] or moving phantoms [17]. They showed that edge-enhancing kernels can reduce the blurring that occurs close to the boundaries of the stents, giving a sharper delineation of the stent wall, less blooming and less artificial in-stent lumen narrowing [10–13, 16, 17, 30].

On the other hand, *in vivo* studies that quantitatively assessed the effect of CT kernels on coronary artery stent blooming artifacts are scarce [18–20, 32]. Hong et al. [18] and Seifarth et al. [20] assessed artificial coronary stent lumen narrowing using 16-slice scanners and image reconstruction with both sharp (B46f) and medium-smooth (B30f) kernels. Both studies [18, 20] showed less blooming artifacts with the sharp kernel, despite a small sample size of 26 and 15 stents, respectively. In a 64-slice scanner study on 71 stents, Cui et al. [32] also demonstrated less blooming with a sharp kernel (HD-detail kernel). In a 256-slice scanner study using a CT pixel attenuation profile method on 28 stents, Oda et al. [19] measured less blooming artifacts with a sharp kernel (CD), in comparison to a soft (CB) kernel. Of note, the observers in the study of Cui et al. [32] were blinded to the type of reconstruction kernel, in contrast to former studies [18–20]. However, in the study of Cui et al. [32], stent blooming quantification was performed by comparing the stent inner diameter to the nominal stent diameter. As mentioned by the authors, the true *in vivo* stent diameter depends on the vessel shape, the dilatation pressure during the stenting procedure, with possible recoil after placement, and does not necessarily correspond to the nominal stent diameter provided by the manufacturer [32]. In our study, we used blinded observers with randomized assignment to kernel measurement. Furthermore, our methodology involved direct measurement of the stent wall thickness, with comparison to the manufacturer stent wall thickness, which is a reliable surrogate of the true *in vivo* stent wall thickness. Of note, our study is also the largest clinical 256-slice CT study assessing the effect of reconstruction kernels on coronary stent blooming.

Earlier *in vivo* studies [17, 33] also assessed in-stent blooming using qualitative visual score analysis, and showed less in-stent blooming with sharp reconstruction kernels. The present *in vivo* study used blinded assessment, and confirms that the use of an edge-enhancing reconstruction kernel is associated with an important improvement in subjective image quality and reduces coronary stent blooming artifacts. Proximal stents, which are large diameter stents, were associated with higher scores of image quality. CT image reconstruction with an edge-enhancing kernel should be considered for the assessment of coronary stents, in addition to a standard kernel, especially for smaller stents.

Our results show a high interobserver variability in the subjective visual grading of in-stent lumen and artifacts, as well as in the quantitative assessment of stent wall thickness. Although less subjective than visual grading, quantitative assessment nevertheless involves human factors, such as the manual placement of an electronic caliper and manual correction for semi-automatic segmentation. Most of all, observer adjudication involves interpretation of ill-defined boundaries of the stent walls due to blooming, called the halo effect [10].

There are limitations to this study. Data from our study show that using conventional filtered back projection as the reconstruction method, an edge-enhancing kernel allows significantly less stent blooming and better image quality than a standard smooth kernel. Iterative reconstruction is a novel reconstruction method which reduces image noise [34] and can improve stent imaging [35–37]. Further studies could investigate the specific impact of iterative reconstruction on the effect of sharp versus smooth kernels in stent CT imaging. Other limitations are that our CT results were not correlated to catheter coronary angiography, and the study did not assess the impact of image reconstruction kernels on the accuracy of in-stent restenosis diagnosis.

## Conclusion

In vivo 256-slice CT assessment of coronary stents shows that image reconstruction with an edge-enhancing kernel significantly reduces stent artifacts, as shown by a reduced stent wall thickness, less overestimation from nominal wall thickness, and better visual scores of in-stent image quality.
